# Supplementary material for: Structural and functional analysis of the Mycobacterium tuberculosis MmpS5L5 efflux pump presages increased bedaquiline resistance
Source: Proc Natl Acad Sci U S A. 2025 Sep 23;122(39):e2516660122. doi: 10.1073/pnas.2516660122 (PMC12501195; doi:10.1073/pnas.2516660122)
Supplement: Supplementary file 1 — Appendix 01 (PDF) [file pnas.2516660122.sapp.pdf]

PNAS

Supporting Information for

Structural and functional analysis of the *Mycobacterium tuberculosis* MmpS5L5 efflux pump presages increased bedaquiline resistance

Adam J. Fountain, Jan Böhning, Stephen H. McLaughlin, Tomos E. Morgan, Paul H. Edelstein, Mark Troll, Meindert H. Lamers, Tanmay A. M. Bharat, Ben F. Luisi*, Lalita Ramakrishnan*

Lalita Ramakrishnan

Email: lr404@cam.ac.uk

Ben Luisi

Email: bfl20@cam.ac.uk

This PDF file includes:

Supporting text

Figures S1 to S20

Tables S1 to S3

SI References

Supporting Information Text

Supplementary materials and methods.

Compounds

The compounds used in MIC assays were as follows: Bedaquiline (Astatech #43211, CAS No. 843663-66-1), Clofazimine (Toku-E #C201, CAS No. 2030-63-9), PBTZ-169 (MedChemExpress #HY-12903, CAS No. 1377239-83-2), OPC-167832 (MedChemExpress #HY-134940, CAS No. 1883747-71-4), TBAJ-587 (MedChemExpress #HY-111747, CAS No. 2252316-16-6), A23187 (Sigma-Aldrich #C7522, CAS No. 52665-69-7), Nigericin (Sigma-Aldrich #SML1779, CAS No. 28643-80-3). TBAJ-876 was a gift from Matt McNeil. All compounds were dissolved in anhydrous DMSO and stored as aliquots at -70°C except for nigericin which was dissolved in a 1:1 solution of DMSO and ethanol.

Complementation and expression plasmid construction

pFLAG-attP-Kan was constructed by in vivo assembly (IVA) cloning (1) by combining a fragment of pFLAG-attP (Addgene #110095) amplified using primer pair P298/P299 and the Kan cassette amplified from pMINTC3GH (Addgene #110077) using primer pair P300/P301. pMINTF3 was constructed by IVA cloning by combining a fragment of pFLAG-attP-Kan amplified using primer pair P721/P722 and the pMINT backbone from pMINTC3GH (Addgene #110077) using primer pair P723/P724. The pMEXC3GF expression vector was generated by replacement of the His tag of pMEXC3GH (Addgene #110082) with a FLAG tag using Q5 mutagenesis (NEB #E0554S) using primer pair P465/P466. Open reading frames were cloned into expression/complementation vectors by FX cloning as previously described (2, 3).

Construction of MmpS5L5 mutants

Mutations in MmpS5L5 containing single/double mutations were constructed by in-vivo assembly (1) using PCR-amplified pINIT-Cat *Mtb MmpS5L5* lacking the replacement region with ~500 bp synthetic DNA fragments containing the desired sequence change (IDT eBlocks). For making MmpS5L5 deletion constructs, pINIT-Cat *Mtb MmpS5L5* was amplified with primers P366/P367 (Δ*MmpS5*), P368/P369 (ΔCoiled-coil), followed by DpnI digestion, phosphorylation with T4 polynucleotide kinase and blunt-end ligated with T4 DNA ligase. MmpS paralog-MmpL5 fusions were constructed using IVA cloning: pINIT-Cat *Mtb MmpS5L5* was amplified with primer pair P367/P583. MmpS paralogs with overhangs were

amplified from Mtb H37Rv genomic DNA using primers P577/P578 (MmpS1), P579/P580 (MmpS2), P581/P582 (MmpS4). All clones were sequenced by whole-plasmid sequencing (Plasmidsaurus).

Electroporation of mycobacteria

Electrocompetent mycobacteria were prepared by 3 washes in 10% glycerol + 0.05% Tween 80. *M. smegmatis* cells were prepared on ice at 4 °C, *M. marinum* and *M. tuberculosis* cells were prepared at room temperature. ~500 ng of plasmid DNA was added to washed bacterial cell suspensions and incubated for 2 minutes. Electroporation was carried out in a 2 mm gap cuvette with electroporation using a BTX^{TM} ECM 630 electroporation system (Settings: 2.5kV, 1000 Ω, 25 µF). 1 ml prewarmed 7H9 + OADS + 0.05% (v/v) Tween 80 was added to the cuvette and incubated at 37ºC for 4 hours (*M. smegmatis*), 16 hours (*M. marinum*) and 24 hours (*M. tuberculosis*) before plating on 7H10 + appropriate antibiotics and incubating at 33ºC (*M. marinum*) or 37ºC (*M. tuberculosis* and *M. smegmatis*). For pFLAG-attP-Kan, pMA-Int (Addgene #110096) was co-electroporated to supply mycobacteriophage L5 integrase for genome integration.

Purification of MmpL5 coiled-coil domain (492-683)

E. coli codon-optimised N-terminally His-TEV tagged MmpL5-CC (residues 492–683) was expressed in *E. coli* BL21(DE3) as inclusion bodies. Bacteria were grown in 2×TY media supplemented with 100 µg ml^{-1} Ampicillin. Expression was induced at OD_{600} 0.7 by addition of 1 mM isopropyl-β-d-thiogalactoside (IPTG), followed by incubation at 37 °C for 3 hours. Bacteria were collected by centrifugation at 5,000 ×g. Bacterial pellets were suspended in 100 ml lysis buffer (50 mM Tris pH 7.6, 150 mM NaCl, 10 mM EDTA, 10 mM DTT, 1% (v/v) Triton X-100, 0.2 mM PMSF supplemented with a spatula tip of Lysozyme, and 500 units Benzonase nuclease (Sigma). Bacteria were lysed by 2 passes through a microfluidizer M110-P at 25 kPsi. Inclusion bodies were pelleted by centrifugation at 20,000 ×g and washed by resuspension with 80 ml lysis buffer using a Potter-Elvehjem homogeniser (GPE scientific limited, #22010). Inclusion bodies were pelleted by centrifugation at 20,000 ×g, and stored at -20 °C. Inclusion body was solubilised with 20 ml 50 mM Tris pH 7.6, 150 mM NaCl, 6 M Urea, 10 mM DTT and incubated for 1 hour at room temperature. Insoluble material was removed by centrifugation at 20,000 ×g. DTT was removed by buffer exchange using a Zeba 7,000 MWCO desalting column equilibrated in unfolding buffer (50 mM Tris pH 7.6, 150 mM NaCl, 6 M Urea). Unfolded protein was diluted to a protein concentration of 1 mg ml^{-1} in unfolding buffer before refolding by slow dropwise

addition to 3 L refolding buffer (50 mM Tris pH7.6, 150 mM NaCl, 1 mM GSSH, 1 mM GSH) with gentle stirring. Refolding was allowed to proceed for 30 minutes at room temperature. Refolded protein was purified by ion-exchange chromatography, using a HiPrep Q 16/10 column pre-equilibrated in IEX A buffer (50 mM Tris pH 7.6, 150 mM NaCl) and then eluted with a linear gradient to 1M NaCl. Peak fractions were concentrated and dialysed overnight against 5 L storage buffer (50 mM Tris pH 8.0, 150 mM NaCl) with a 3 kDa MWCO dialysis cassette. Protein aliquots were flash frozen and stored at -70°C until use.

Circular dichroism spectroscopy

Circular dichroism data were collected using a JASCO J-815 spectropolarimeter. Spectra Manager (1.55) was used for data collection. Circular dichroism spectra were acquired at 14 μM protein concentration in 50 mM Tris pH 7.6, 150 mM NaCl at room temperature. Data were collected in a 1 mm quartz cuvette between wavelengths of 190 nm and 260 nm with the instrument set as follows: band width, 1 nm; scanning speed, 50 nm min^{-1}; response time, 1 s. Each circular dichroism spectrum was obtained by averaging eight scans and subtracting the background signal of the buffer and cuvette. The spectra were converted from ellipticities (mdeg) to mean residue ellipticities ($deg \cdot cm^2 \cdot dmol^{-1} \cdot res^{-1}$) by normalizing for concentration of peptide bonds and the cell path length using the equation:

$MRE = \theta \times 10^6 / c \times l \times n$, where the variable θ is the measured difference in absorbed circularly polarized light in millidegrees, c is the concentration of the protein in micromolar, l is the path length of the cuvette in millimetres, and n is the number of amide bonds in the polypeptide.

Intact mass spectrometry

Purified N-terminally His-TEV tagged MmpL5 coiled-coil domain was analysed by mass spectrometry both before and after incubation at 45 °C for 30 minutes in 0.05 mM TCEP. The protein was analysed by LC-MS using a Vanquish HPLC (Thermo Scientific, USA) with mobile phase A – 90% water, 9.9% acetonitrile with 0.1% formic acid and mobile phase B – 80% acetonitrile, 19.9% water, and 0.1% formic acid. Delivered flow was 300 μl min^{-1}. Proteins were trapped on a MAbPac RP 2.1 x10 mm trapping column for two minutes before separation using a MAbPac RP 2.1 x 50 mm LC column. The gradient was from 10% to 30% B over 2 minutes before increasing to 55% B over the next 8 minutes at 80 °C. Proteins were eluted and ionized with a HESI ion source onto an orbitrap mass spectrometer (Exploris MX, Thermoscientific USA). MS data were acquired in in a broadband detection mode (R = 120,000,

m/z 600 – 2000, Intact protein mode) in positive ion mode (AGC = 100%, µscans = 10, in source energy = 10 eV). Protein deconvolution was carried out using a sliding window Xtract (isotopically resolved) deconvolution to generate monoisotopic protein masses.

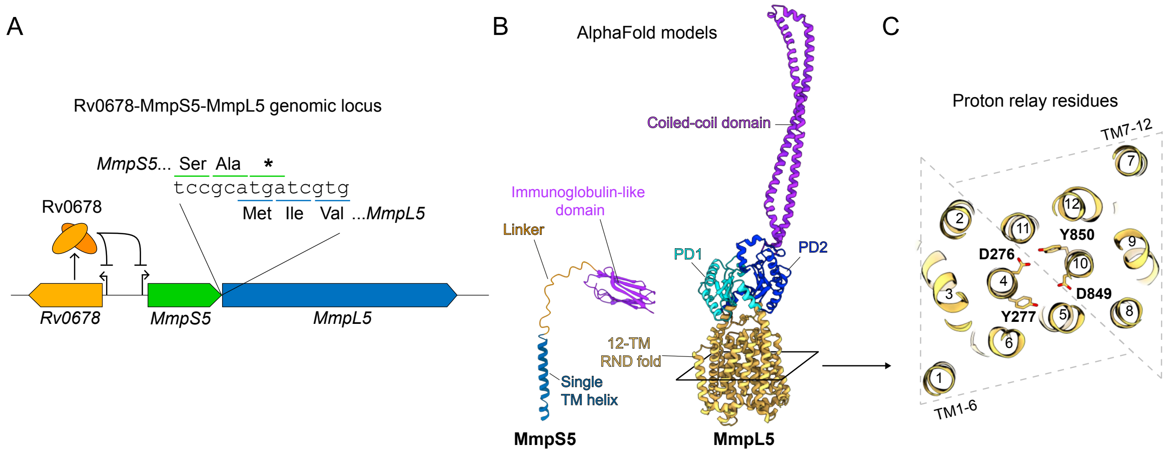

D

Strain	Bedaquiline MIC (ng ml^{-1})	Clofazimine MIC (ng ml^{-1})	PBTZ-169 MIC (ng ml^{-1})	TBAJ-876 MIC (ng ml^{-1})	TBAJ-587 MIC (ng ml^{-1})	OPC-167832 MIC (ng ml^{-1})	A23187 MIC (µg ml^{-1})	Nigericin MIC (µg ml^{-1})
Number of replicates	3	3	3	2*	3	4	4	4
mc^{2}6206	25.0	7.8	0.78	12.5	6.25	0.19	2.0	1.0
mc^{2}6206 Δ*S5L5*	6.25	1.95	0.39	3.12	1.56	0.19	0.5	0.5
mc^{2}6206 Δ*S5L5* + Mtb *MmpS5L5*	50.0	7.8	1.56	12.5	6.25	0.79	8.0	2.0
mc^{2}6206 Δ*S5L5* + Mtb *MmpS5L5* Y277F	50.0	7.8	0.39	12.5	6.25	N.D	N.D	N.D
mc^{2}6206 Δ*S5L5* + Mtb *MmpS5L5* D849N	6.25	1.95	0.39	3.12	1.56	N.D	N.D	N.D
mc^{2}6206 Δ*S5L5* + Mtb *MmpS5L5* Y850F	6.25	1.95	0.39	3.12	1.56	N.D	N.D	N.D

* - Not in triplicate due to limited reagent

Fig. S1: MmpS5L5 is a multidrug efflux pump

(A) Diagram of the Rv0678 MmpS5 MmpL5 genomic locus. MmpS5 and MmpL5 are translationally coupled with overlapping stop-start codons. (B) AlphaFold models of *M. tuberculosis* MmpS5 (Uniprot: P9WJS7) and MmpL5 (Uniprot: P9WJV1) with structural features annotated. (C) View of slice through the transmembrane domain of MmpL5, with TM helices numbered. In TMs 4 and 10, Asp-Tyr 'Proton-relay' residues are shown. (D) MIC values for knockout, complemented strains performed in biological triplicate. MICs are given in either ng ml^{-1} or µg ml^{-1}.

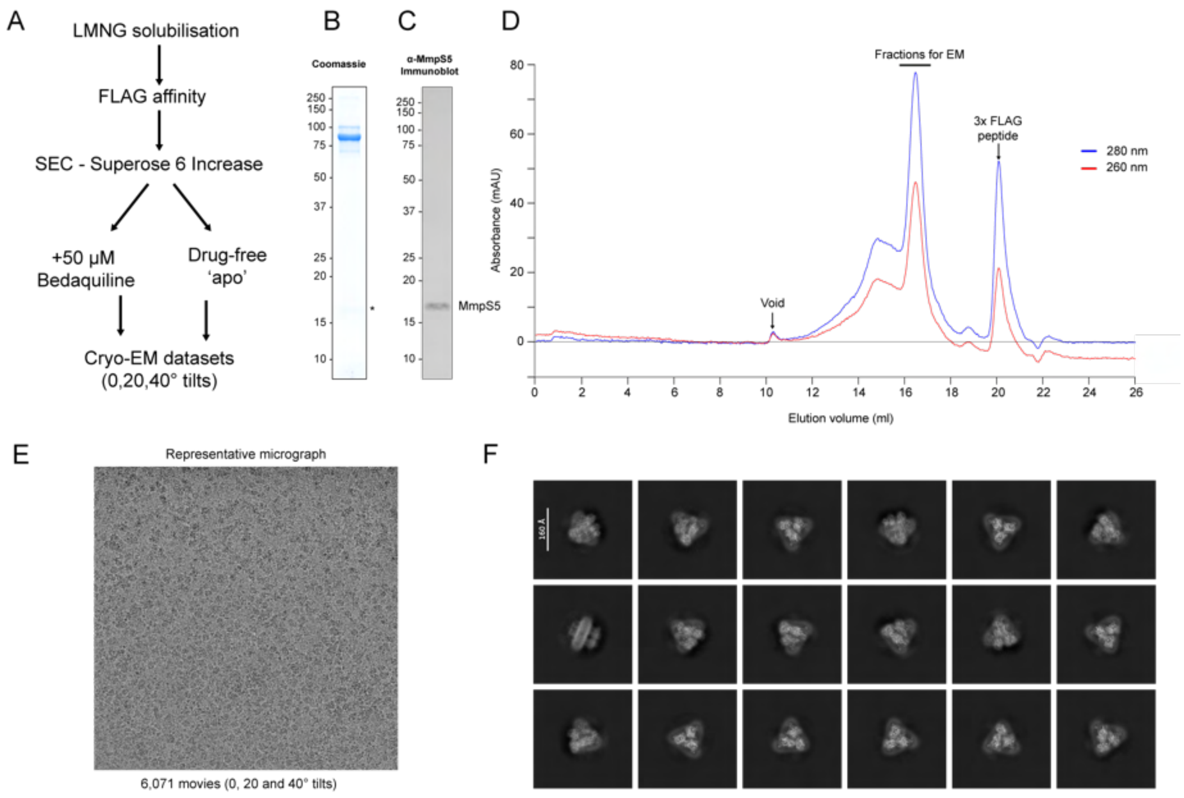

Fig. S2: Purification of MmpS5L5 ΔCC

(A) Purification scheme for MmpS5L5-GFP-FLAG. (B) Coomassie-stained SDS-PAGE gel of peak fraction used for cryo-EM sample preparation. (C) Anti-MmpS5 immunoblot of fractions used for cryo-EM sample preparation. (D) Size-exclusion chromatography profile of affinity purified MmpL5-GFP-FLAG on a Superose 6 10/300 Increase column. (E) Representative cryo-EM micrograph of the +bedaquiline dataset. (F) 2D class averages for the trimeric species of MmpL5 in the dataset.

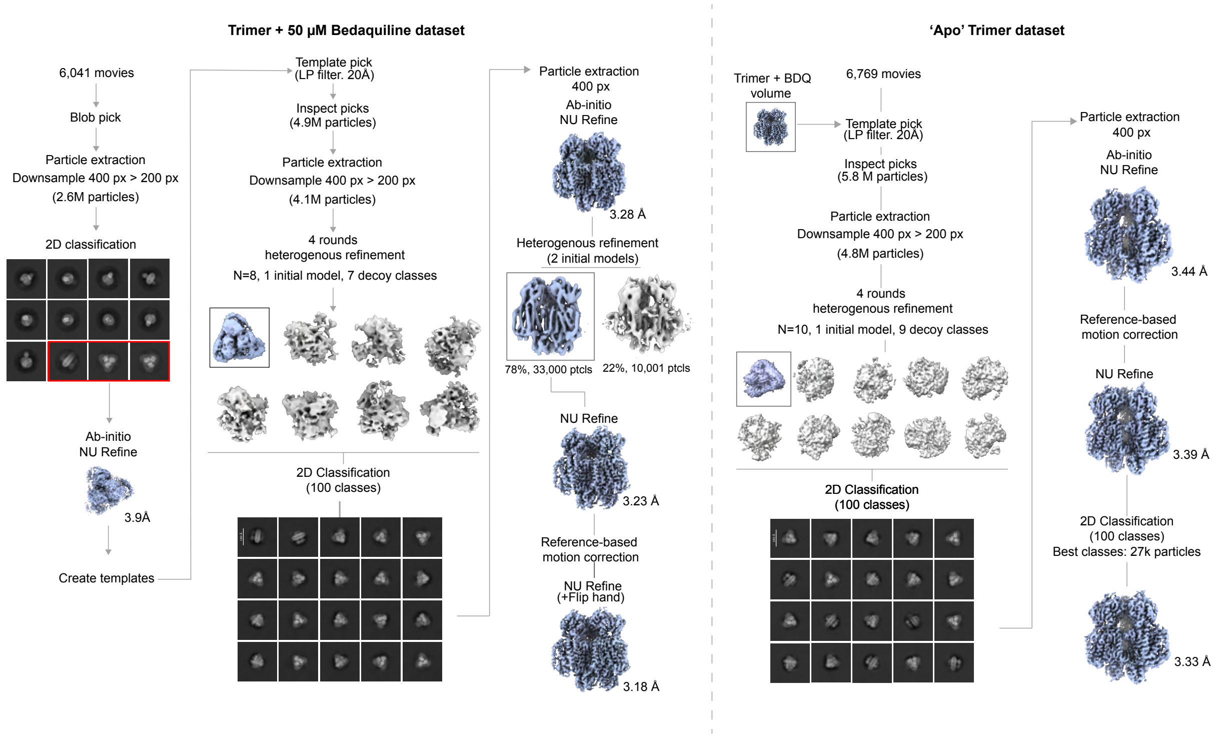

Fig. S3: Cryo-EM processing pipeline

Cryo-EM processing workflow (cryoSPARC) for both apo and bedaquiline-incubated trimer datasets.

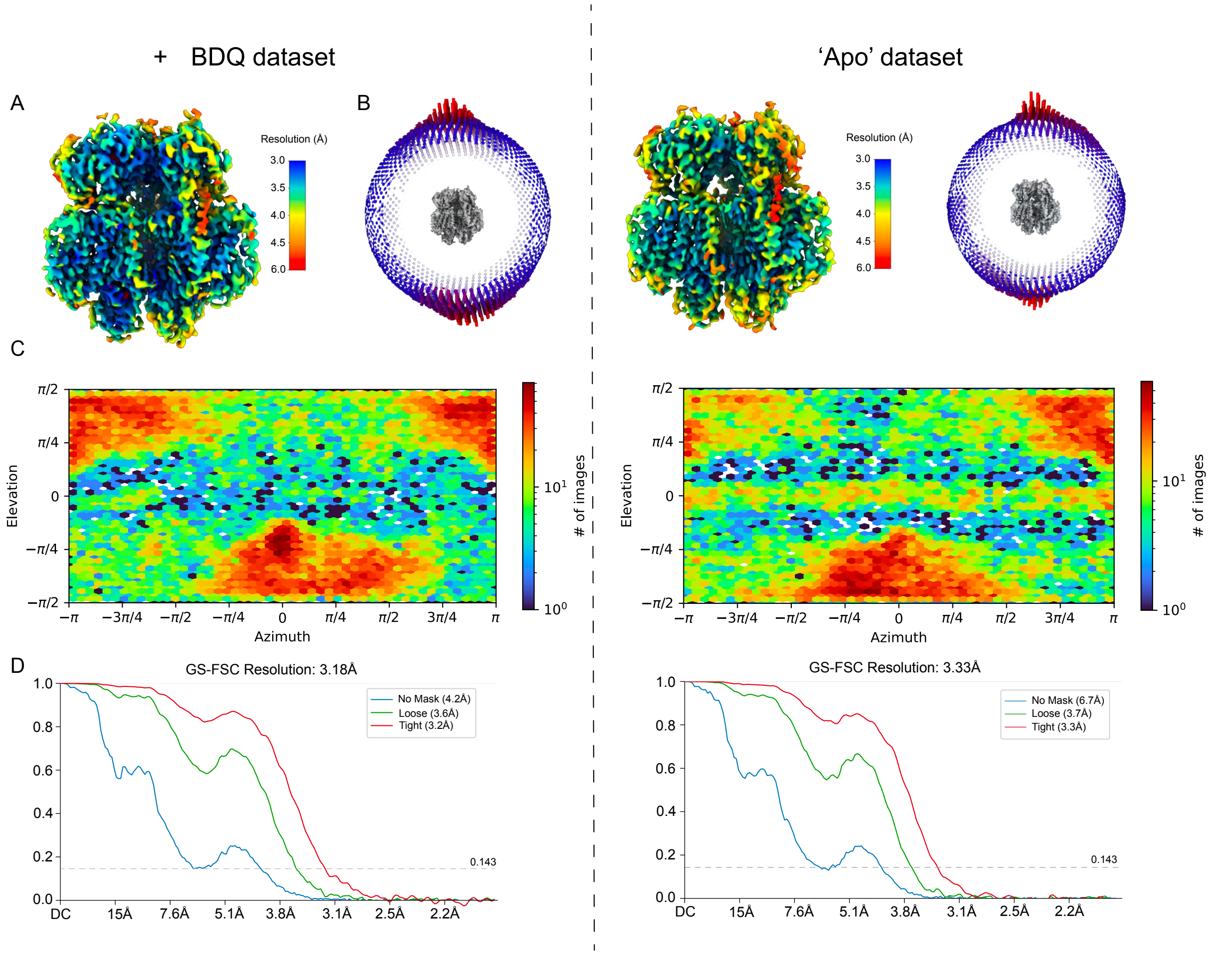

Fig. S4: Local resolution, angular distributions, and FSC curves.

(A) Cryo-EM density map coloured according to local resolution. TM helix 2 and PD2 have lower resolution, consistent with flexibility in these regions. (B–C) Angular distributions of the final reconstruction. (D) Fourier shell correlation (FSC) plot of the trimer reconstruction.

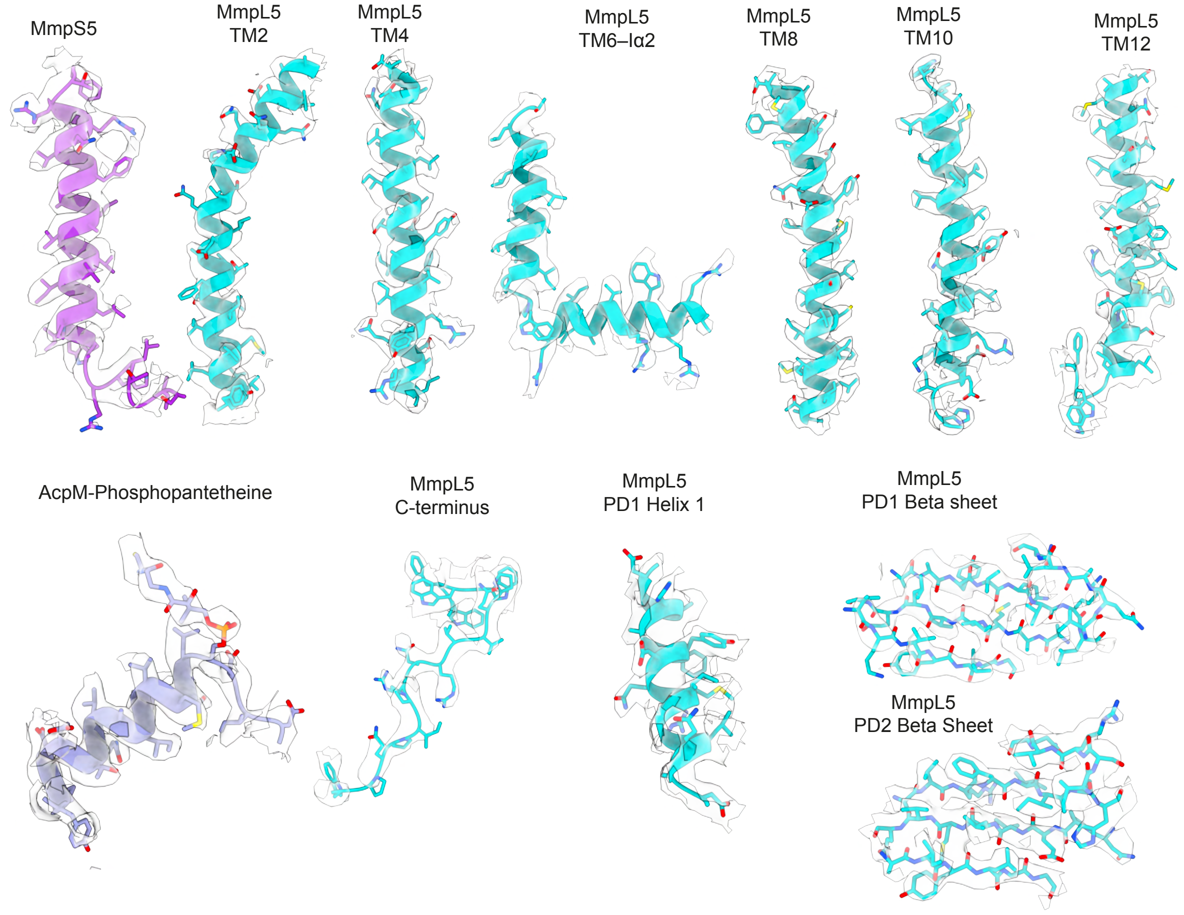

Fig. S5: Model-map fit for MmpS5, MmpL5 and AcpM

Representative map-model fit for cryo-EM densities from the bedaquiline-incubated trimer map. Density was extracted using ChimeraX's 'volume zone' tool with a 2.0–3.0 Å distance cutoff around the indicated secondary structure.

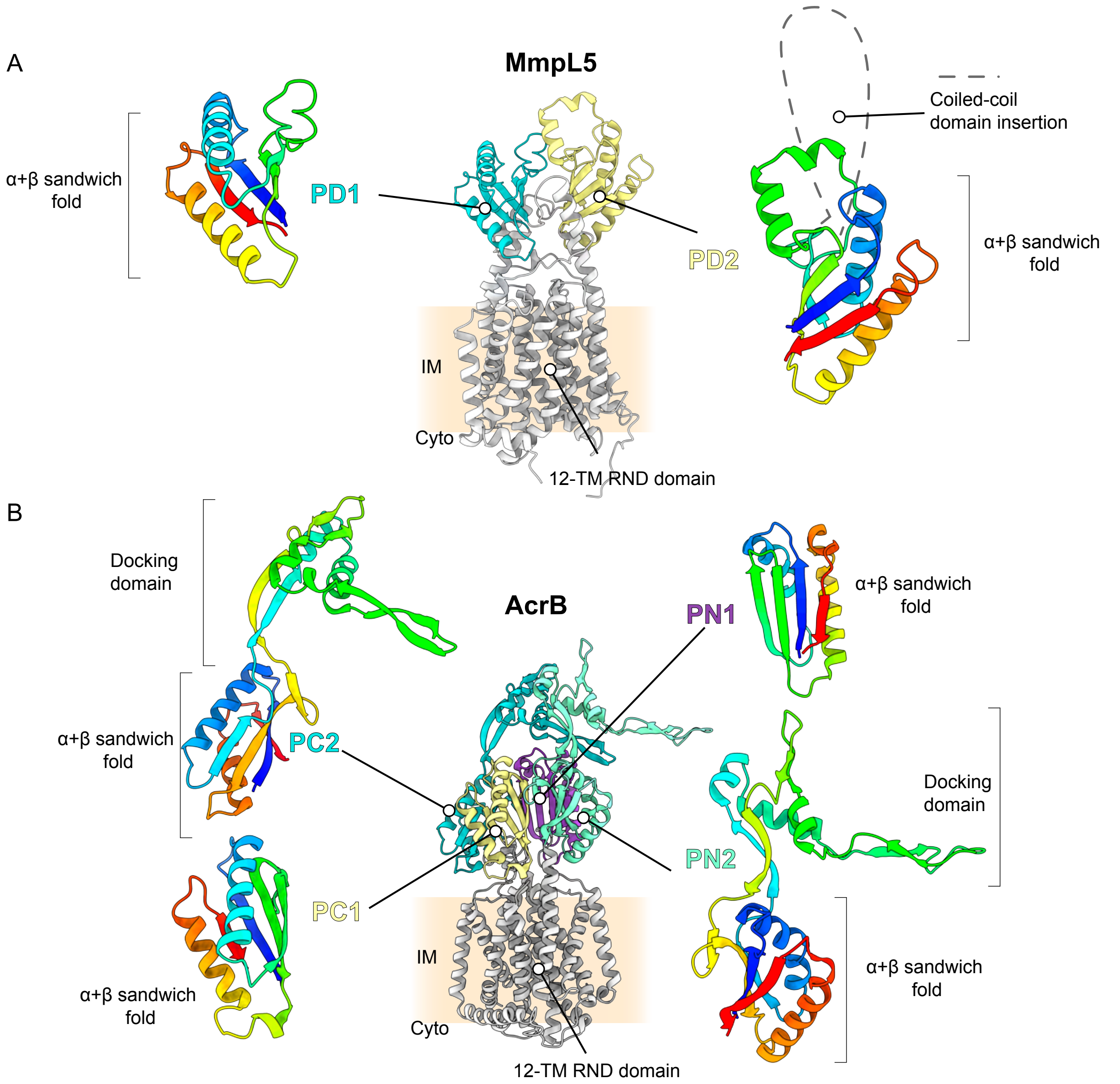

Fig. S6: Comparison of AcrB and MmpL5

Upper, Domain structure of *Mtb* MmpL5 ΔCC. The coiled-coil domain inserted into PD2 is shown as a dashed line. Lower, Domain structure of an *E. coli* AcrB protomer. Individual domains are coloured with a rainbow gradient from blue at the N-terminus to red at the C-terminus.

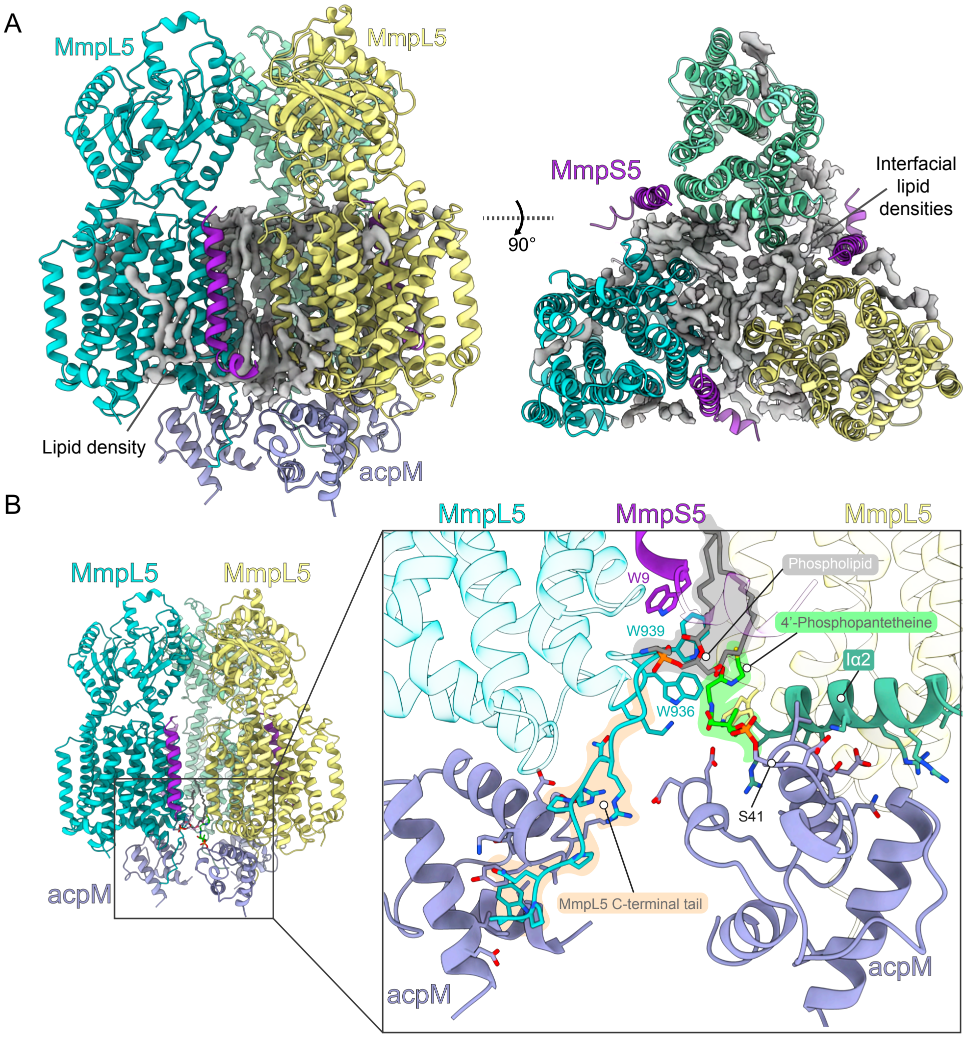

Fig. S7: Lipids and AcpM stabilise intersubunit interactions

(A) Structural model of MmpS5L5-AcpM with unassigned lipid-like densities (grey) at the interfaces between subunits. (B) Interface between protomers at the inner leaflet of plasma membrane. AcpM Ser41 is post-translationally modified with 4'-Phosphopantetheine (green). A phospholipid (grey) interacts with Trp9 of MmpS5 (purple). AcpM (lilac) interacts with MmpL5's intracellular Iα2 helix (teal), and MmpL5's C-terminal tail (blue/orange).

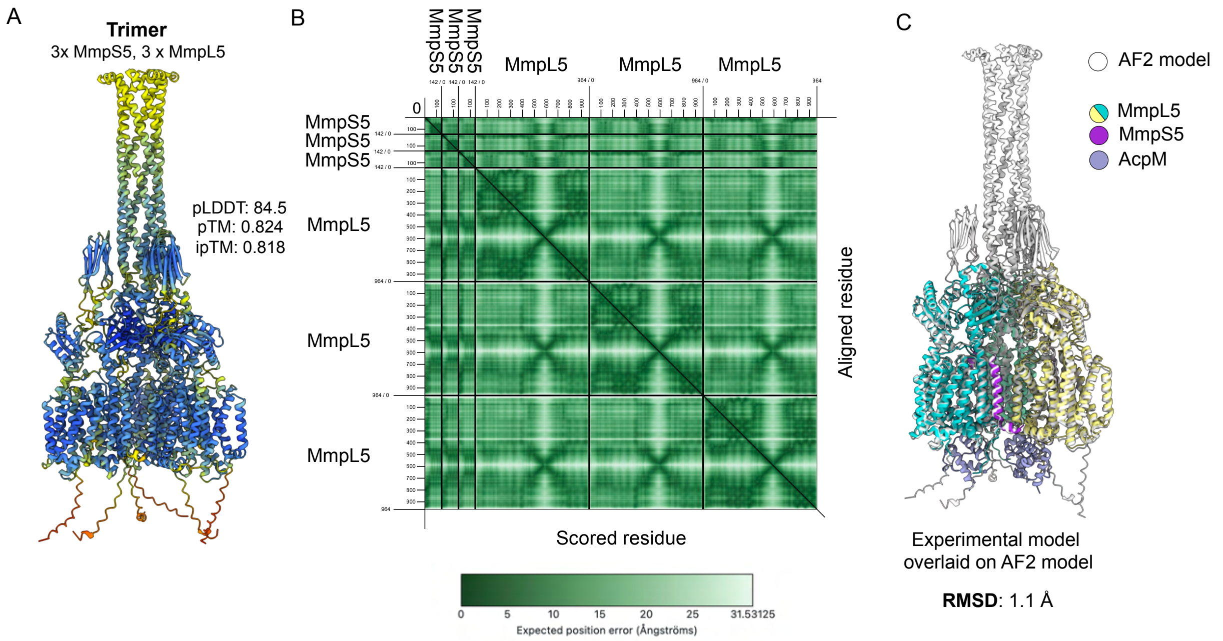

Fig. S8: Alphafold2 prediction of a homotrimer of MmpS5 and MmpL5

(A) Structural model of a MmpS5L5 trimer. Models are coloured by per-residue pLDDT score. The pTM and ipTM values for the corresponding model are indicated. (B) Predicted aligned error (PAE) plot of the trimeric model. (C) Overlay of the experimentally determined structure of MmpS5L5-AcpM, on the AlphaFold2 model. RMSD – Root mean square deviation.

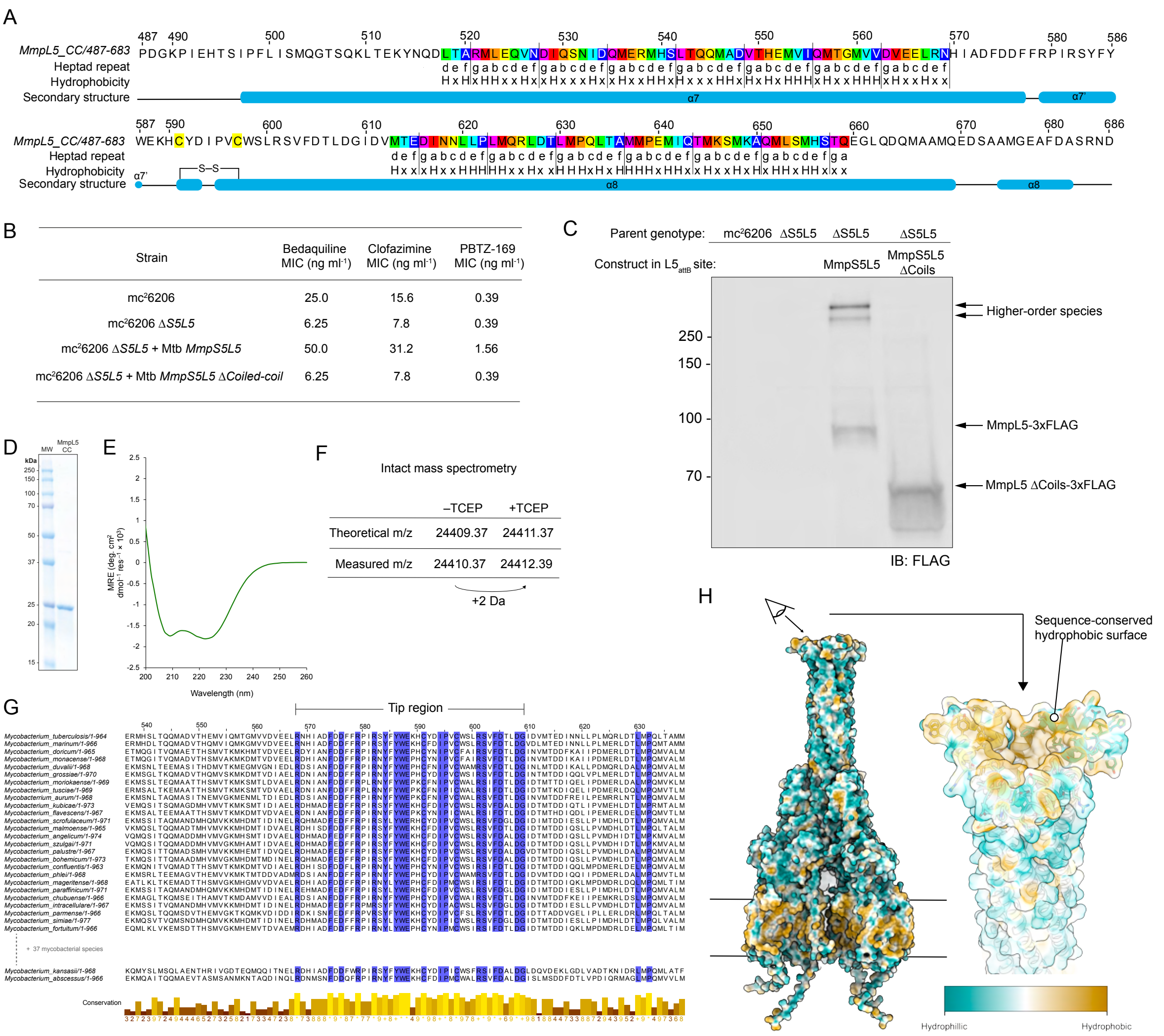

Fig. S9: Coiled-coil heptad repeat sequence analysis and functional validation.

(A) Annotated amino acid sequence of *M. tuberculosis* MmpL5's coiled-coil domain (residues 487 – 683). Hydrophobic amino acids are indicated with "H". Non-hydrophobic amino acids are indicated with 'x'. Heptad repeats are classified according to (4). *gabcef* indicates the position within the heptad repeat. S–S disulfide bond. (B) Bedaquiline, clofazimine and PBTZ-169 MIC values for strains lacking residues (494–687) (ΔCC). (C) anti-FLAG immunoblot showing that the ΔCC construct is expressed. (D) Coomassie stained SDS-PAGE gel of purified MmpL5 coiled-coil domain. (E) CD spectrum of MmpL5 coiled-coil domain. (F) Theoretical and measured monoisotopic masses of MmpL5 coiled-coil domain, with and without TCEP reduction. (G) Multiple sequence alignment of MmpL5 from a wide sampling of mycobacterial species. Residues 537-638 are shown. 100% sequence conserved amino acids are

coloured blue. Conservation score is shown below. (H) Surface of the MmpS5L5 Alphafold2 model coloured according to the Kyte-Doolittle hydrophobicity scale (5).

A

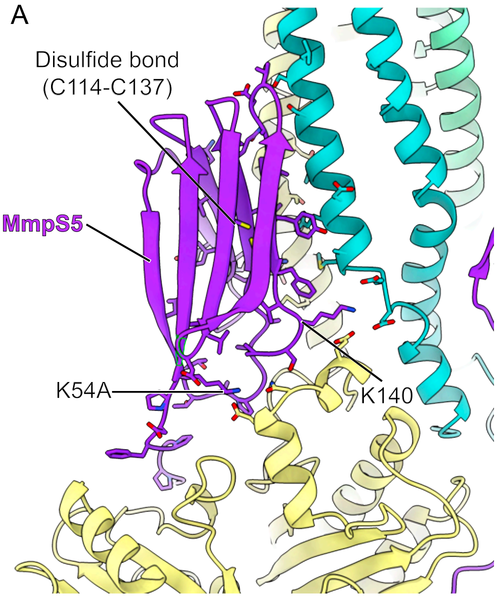

B

Strain	Bedaquiline MIC (ng ml^{-1})	Clofazimine MIC (ng ml^{-1})	PBTZ-169 MIC (ng ml^{-1})
mc^{2}6206	25.0	15.6	0.39
mc^{2}6206 Δ*S5L5*	3.1	7.8	0.39
mc^{2}6206 Δ*S5L5* + Mtb *MmpS5L5*	50.0	31.2	1.56
mc^{2}6206 Δ*S5L5* + Mtb *MmpL5* only	3.1	7.8	0.39
mc^{2}6206 Δ*S5L5* + Mtb *MmpS5* K54A *MmpL5*	6.3	7.8	0.39
mc^{2}6206 Δ*S5L5* + Mtb *MmpS5* K140A *MmpL5*	25.0	7.8	0.79
mc^{2}6206 Δ*S5L5* + Mtb *MmpS5 K54A K140A MmpL5*	3.1	7.8	0.39
mc^{2}6206 Δ*S5L5* + Mtb *MmpS5 C137S MmpL5*	3.1	7.8	0.39

C

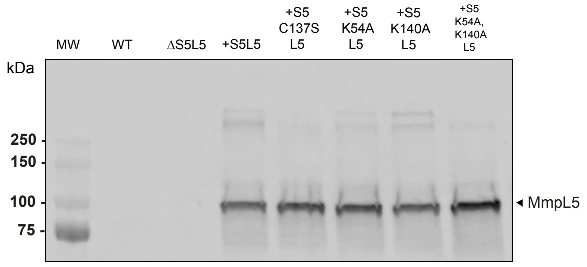

Fig. S10. Mutations at the S5-L5 interface prevent complementation

(A) Structural model of the MmpS5-MmpL5 interface from the AlphaFold2 prediction. (B) Bedaquiline, clofazimine and PBTZ-169 MIC values for selected MmpS5 mutations. (C) Anti-FLAG immunoblot showing MmpL5 expression in all strains.

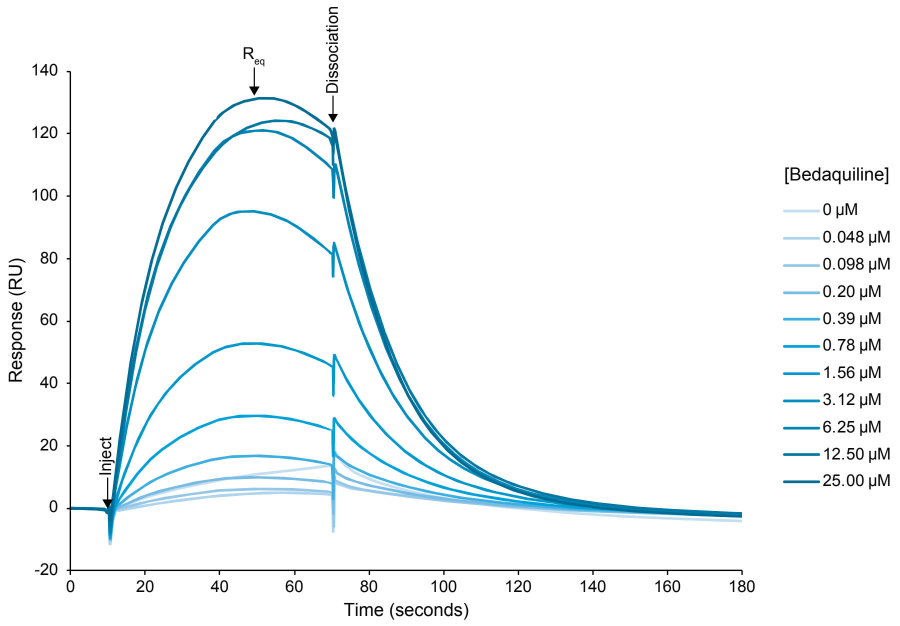

Fig. S11. Representative SPR sensorgram of bedaquiline binding to *Mtb* MmpL5 ΔCC-GFP

Sensorgram from a representative surface plasmon resonance (SPR) experiment, showing the binding of bedaquiline to an *Mtb* MmpL5 ΔCC-GFP. The x-axis represents time in seconds, and the y-axis represents the response in resonance units (RU). Different concentrations of bedaquiline, ranging from 0.024 µM to 25.00 µM, were injected over the sensor chip, as indicated by the legend on the right. We have observed that the response begins to decrease before the dissociation phase in multiple independent experiments. The peak of the response is used as the R_{eq} value.

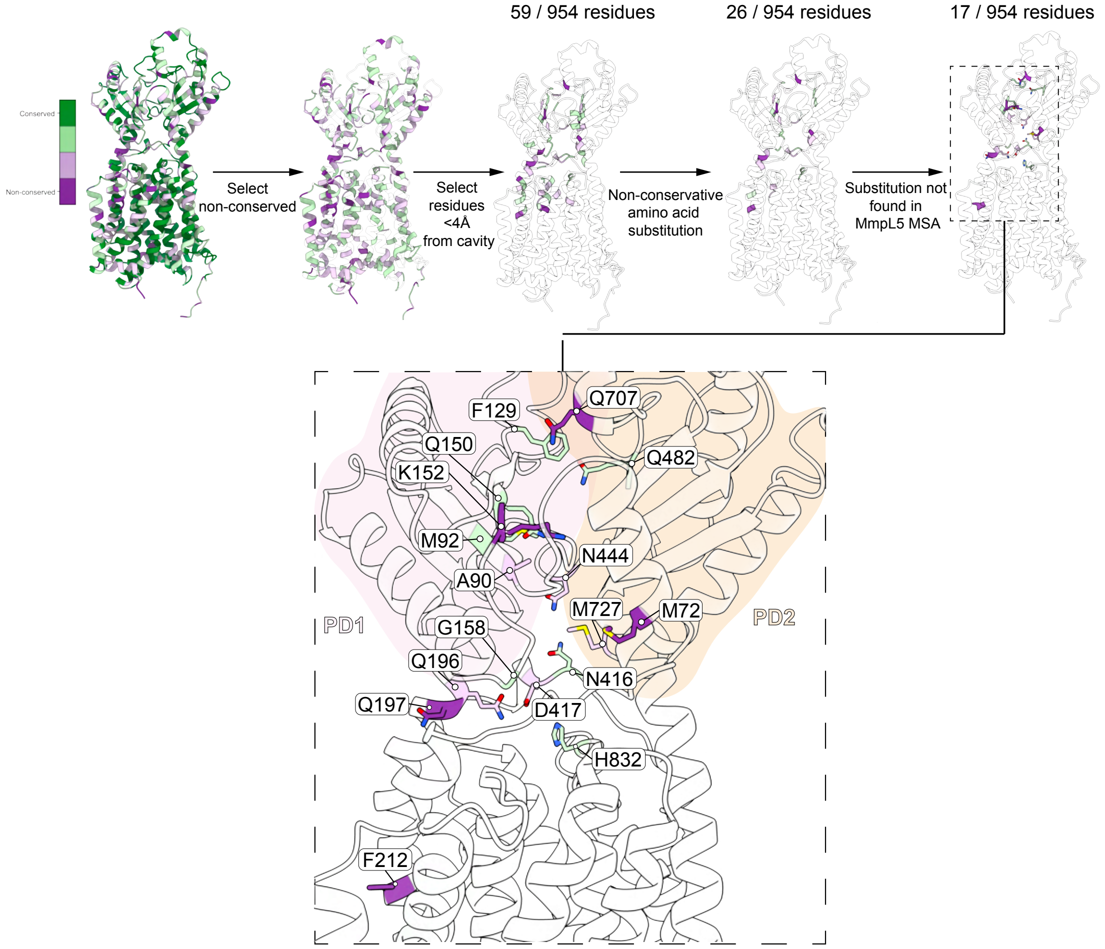

Fig. S12. Paralog-guided mutation selection criteria

(A) Selection criteria for identifying candiate residues with functional importance. Sequence conservation between MmpL5/4/2/1 was mapped onto the structure of MmpL5. 100% sequence conserved residues were discounted. Residues within 4 Å of the cavity formed by PD1, PD2 and TMs 1–4 (orange) were selected. Of these, those with non-conservative amino acid substitutions within an MSA of MmpL5/4/2/1 were selected. Selected substitutions that were found in MmpL5s from other mycobacterial species were discounted. (B) Structural model of MmpL5, showing the positions of selected residues based on the selection criteria.

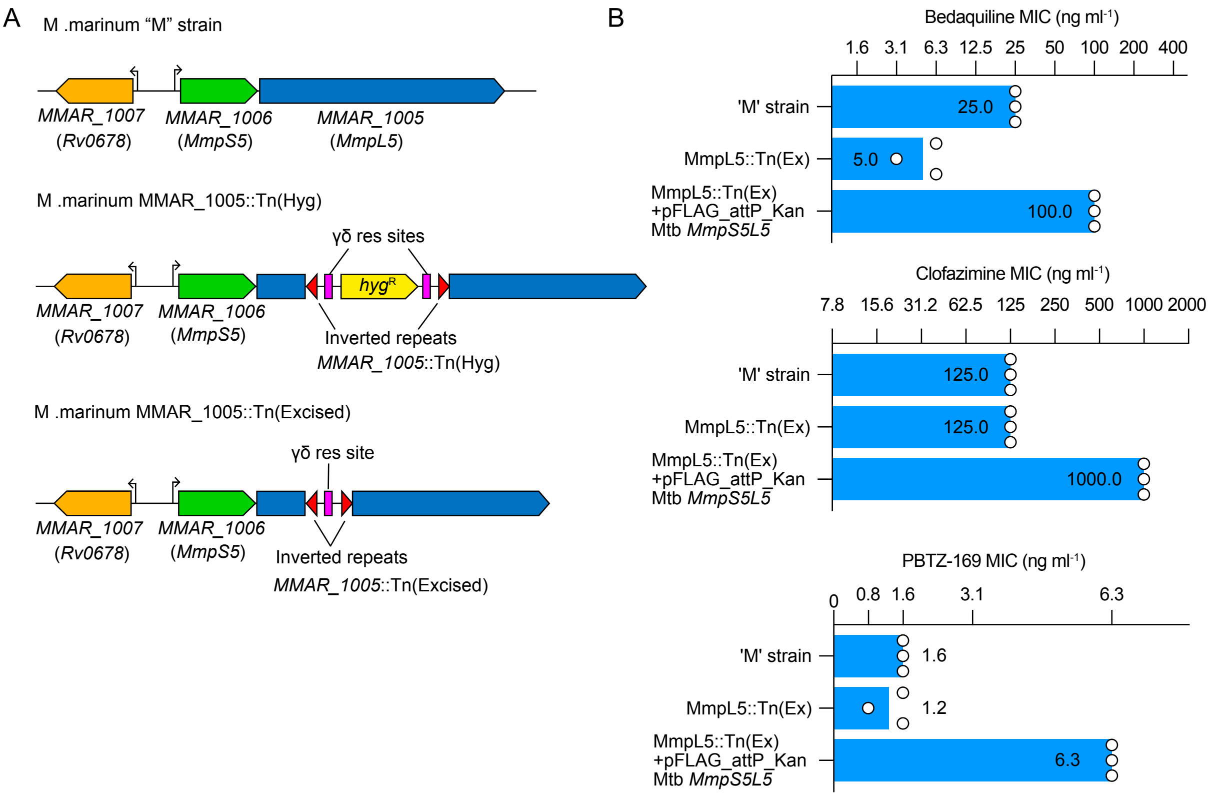

Fig. S13. *M. marinum* is a suitable surrogate for MmpS5L5 phenotypes

(A) Schematic of each *M. marinum* transposon strain used in this study. (B) Bar graphs of MIC values for *M. marinum* strains complemented with pFLAG-attP-Kan *Mtb MmpS5L5* integrated at the $L5_{attB}$ site for bedaquiline, clofazimine and PBTZ-169. Open circles are plotted MIC values for independent repeats. Values for each column indicate the geometric mean of the MIC values in ng ml^{-1}.

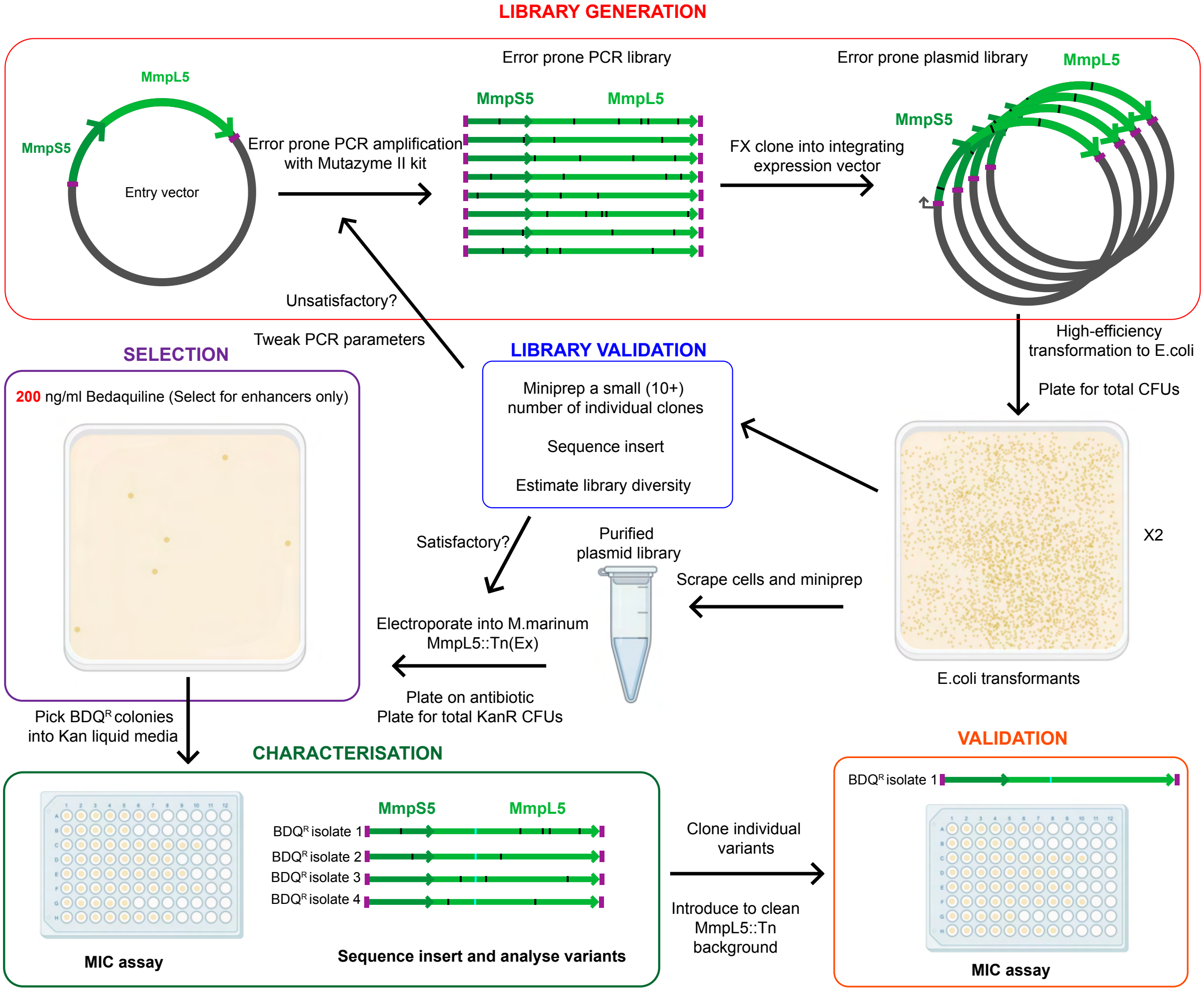

Fig. S14. Error-prone PCR library generation scheme

Outline of error-prone PCR library generation and selection experiments. In the library generation step, the coding sequence is PCR amplified from an FX-compatible entry vector and assembled into the integrative pMINTF3 vector. The library was electroporated into *E. coli*, and plasmid DNA from a subset of colonies was sequenced to estimate mutation rate. Library DNA was isolated from the plate.

Suppressor selection

MmpL5 variant	CFU plated	Number resistant clones	Resistance Frequency
N444K	2.78×10^{9}	12	2.3×10^{-8}

Resistant isolate(s)	Genotype
Sup-1–Sup-4, Sup-8–Sup11	Recombination MMAR_1005::Tn with $L5_{attB}$::pFLAG-attP-Kan *Mtb MmpS5L5* N444K
Sup-5 and 6	$L5_{attB}$::pFLAG-attP-Kan *Mtb MmpS5L5* N444K, Y331D
Sup-7	atpE A63P
Sup-12	atpE A63V

EP-PCR variant selection

Bedaquiline concentration	Total Kan^{R} colonies	BDQ^{R},Kan^{R} colonies	Proportion of library
200 ng ml^{-1}	7.6×10^{4}	5	0.005%

Resistant isolate(s)	Number of mutations	Nucleotide/amino change
BDQ-2	6	*MmpL5*: c.587T>A (p.Val193Asp), c.704T>A (p.Met235Lys), c.1269C>T (p.Ala423Ala), c.1279G>T (p.Ala427Ser), c.1447G>T (p.Ala483Ser), c.2527C>T (p.Leu843Leu)
BDQ-3 and BDQ-5	4	*MmpS5*: c.122A>G (p.Lys42Arg) *MmpL5*: c.587T>A (p.Val193Asp), c.903C>G (Thr301Thr), c.2106A>G (Gln702Gln),
BDQ-6	5	*MmpL5*: c.587T>A (p.Val193Asp), c.945G>A (p.Leu315Leu), c.1087C>A (p.Arg363Arg), c.1162C>T (Arg388Cys), c.1890C>T (p.Leu630Leu),
BDQ-7	4	*MmpL5*: c. 755T>A (p.Phe252Tyr), c.1766A>G (p.Lys587Arg), c.2356C>A (p.Leu786Met), c.2705T>C (p.Val902Ala)

Fig. S15. Summary of resistance selection experiments

Tables summarising the input parameters and outcomes of the suppressor selection (Upper) and EP-PCR variant selection experiments. Sup-# refers to the isolate from the suppressor selection. BDQ-# refers to the isolate from the EP-PCR variant selection.

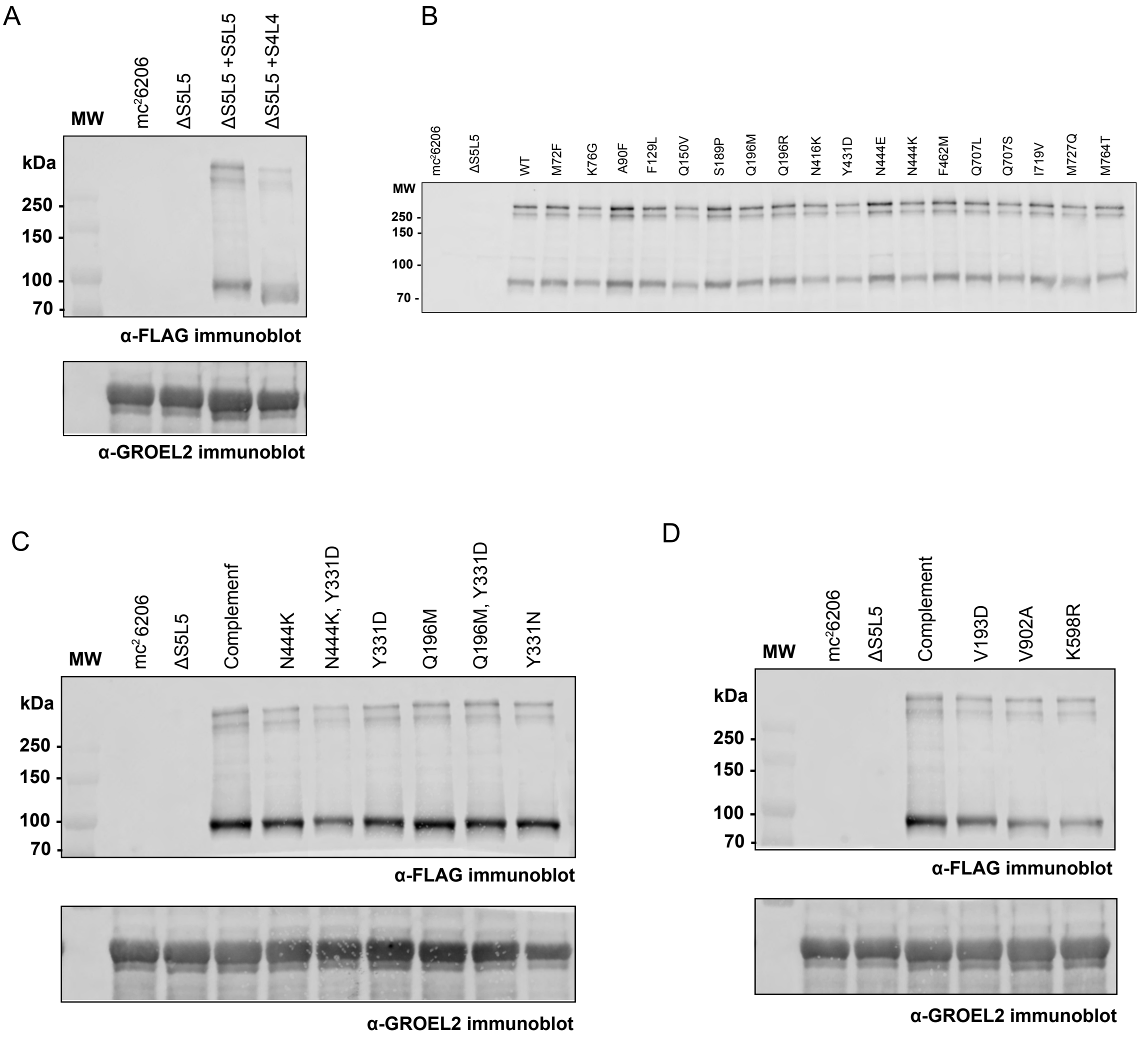

Fig. S16. Immunoblot analysis of MmpS5L5 mutant strains

(A–D) Immunoblot analysis of the strains used in this work. 20 µg total protein was loaded per lane. Upper blots indicate anti-FLAG immunoblot to detect FLAG-tagged MmpL5. Lower blots are loading controls against the cytoplasmic protein GROEL-2.

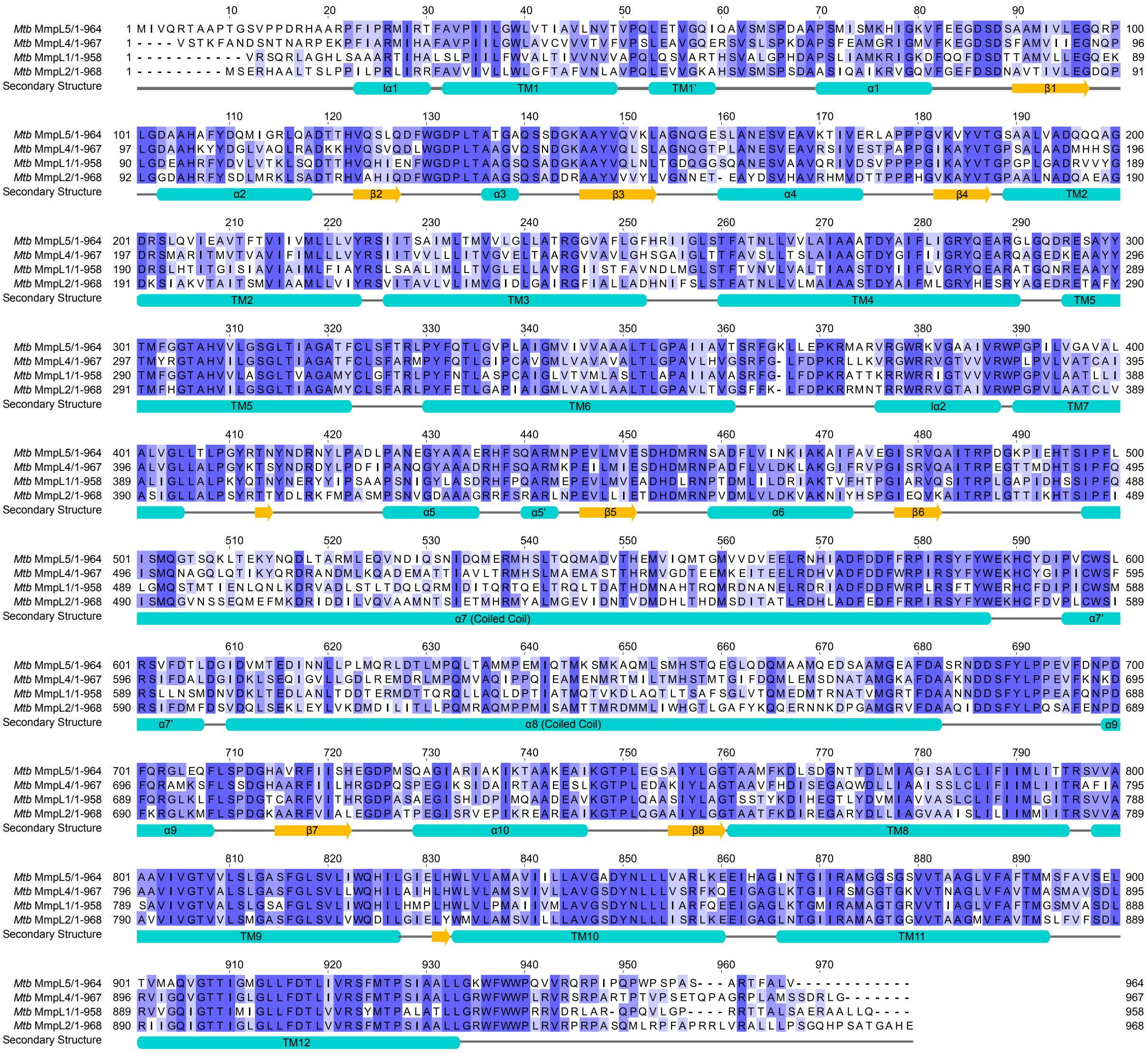

Fig. S17. Multiple sequence alignment of MmpL5/4/2/1

Multiple sequence alignment of MmpL5/4/2/1. Numbering is according to position in MmpL5. Residues are coloured according to strength of sequence conservation. Secondary structure elements are indicated below the alignment.

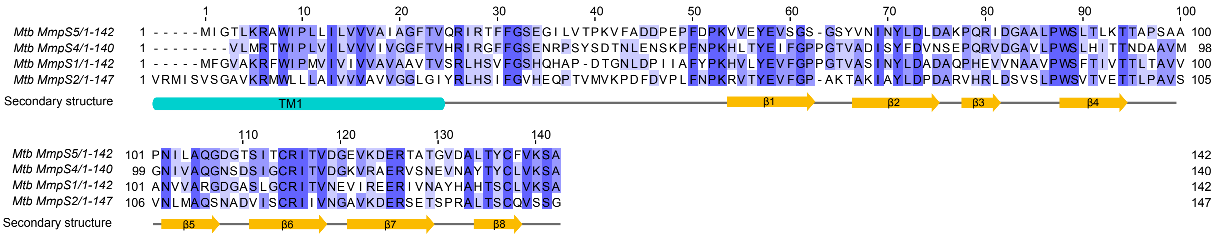

Fig. S18. Multiple sequence alignment of MmpS5/4/2/1

Multiple sequence alignment of MmpS5/4/2/1. Numbering is according to position in MmpS5. Residues are coloured according to strength of sequence conservation. Secondary structure elements are indicated below the alignment.

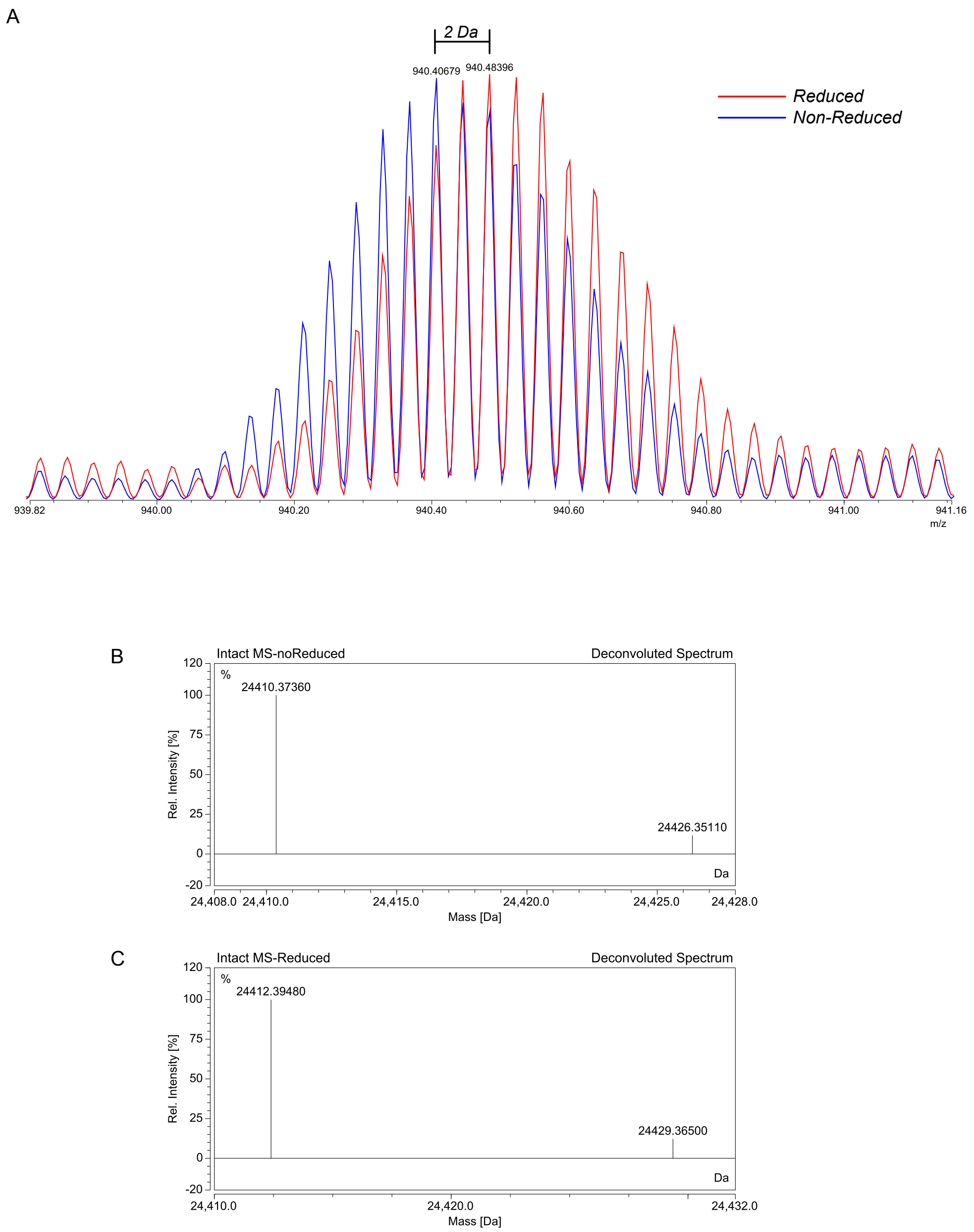

Fig. S19. Mass spectra of purified MmpL5 coiled-coil domain (493–683) under non-reducing and reducing conditions.

(A) Raw mass spectrometry spectra of the MmpL5 coiled-coil domain. The blue trace represents the non-reduced sample, showing a distinct isotopic envelope. The red trace corresponds to the TCEP-reduced sample (B) Deconvoluted mass spectrum of non-reduced MmpL5 coiled-coil domain**.** The primary peak at 24410.37 Da corresponds to the expected mass of the oxidized coiled-coil domain. (C) Deconvoluted mass spectrum of TCEP-reduced MmpL5 coiled-coil domain. The primary peak at 24412.39 Da, approximately 2 Da higher than the non-reduced sample, confirms the presence of an intramolecular disulfide bond that is reduced by TCEP.

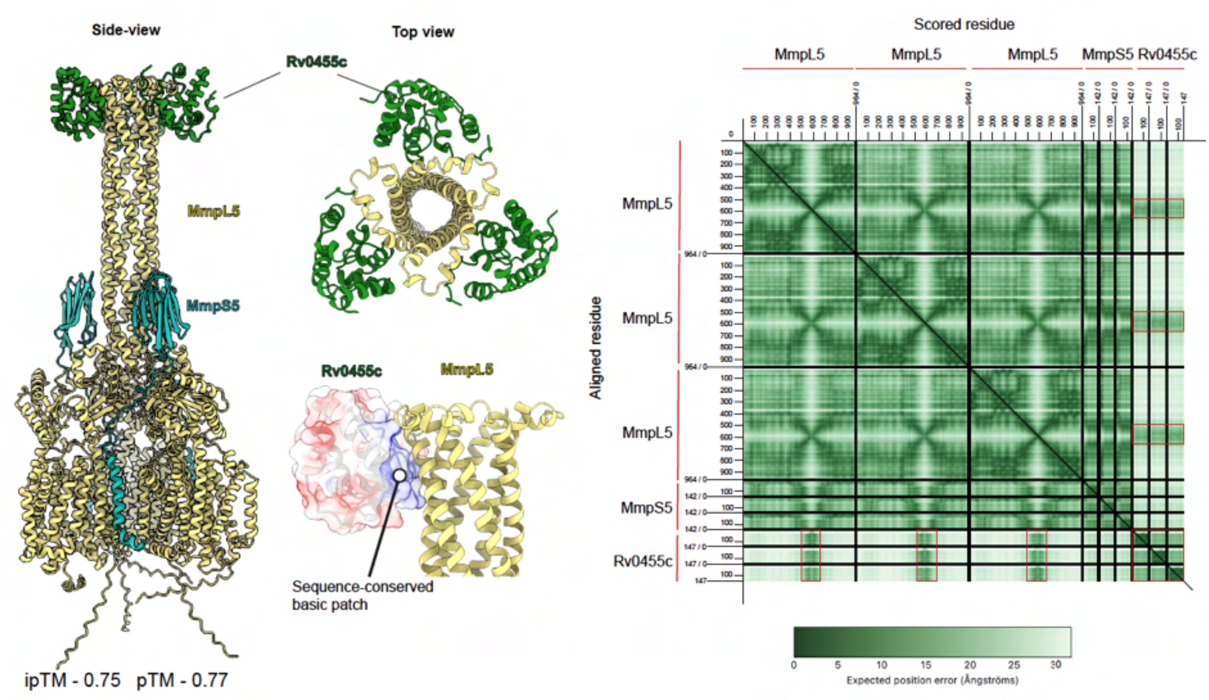

Fig. S20. AlphaFold3 prediction of a 3:3:3 Rv0455c:MmpS5:MmpL5 complex

Rv0455c (green) is predicted to interact with the tip of the coiled coil domain of MmpL5 via a sequence-conserved basic patch. Predicted aligned error (PAE) plot – right.

Table S1. Summary of bacterial strains and constructs used in this study

Bacterial strains

Reagent or resource	Description	Source	Identifier
Organisms/Strains			
E. coli DH5α		Invitrogen	
M. smegmatis mc^{2}155		Snapper *et al*., 1990	
M. tuberculosis H37Rv mc^{2}6206 Δ*panCD* Δ*leuCD*		Vilcheze et al., 2018	
M. tuberculosis H37Rv mc^{2}6206 Δ*panCD* Δ*leuCD* Δ*S5L5*::loxP	Strain containing a deletion of the MmpS5L5 operon, generated by recombineering	Fountain and Waller *et al.* 2025	
M. tuberculosis H37Rv mc^{2}6206 Δ*panCD* Δ*leuCD* Δ*S5L5*::loxP attB$_{L5}$::pMINTF3 *Mtb MmpS5L5*	Complemented ΔS5L5 strain with integrative pMINTF3 vector expressing *Mtb* MmpS5L5.	Fountain and Waller *et al.* 2025	
M. marinum 'M' strain			
M. marinum MMAR::1005::Tn		L. Ramakrishnan lab strain	
M. marinum MMAR::1005::Tn attB$_{L5}$::pFLAG-attP-Kan *Mtb MmpS5L5*		This study	

DNA constructs

Reagent or resource	Description	Source	Identifier
pMEXC3GF	ATc-inducible episomal expression construct with C-terminal GFP-FLAG tag	This study	
pMEXC3GF *Mtb MmpS5L5(*ΔCC)	ATc-inducible episomal expression construct encoding MmpS5 and MmpL5 ΔCC (Δ494–687)	This study	
pET-His6-TEV-MmpL5(492–683)	*E. coli* expression construct of *E. coli* codon-optimised MmpL5 coiled-coil domain	This study	
pINIT-Cat	FX cloning entry vector	Geertsma and Dutzler, 2011	Addgene #46858
pMA-Int	*E. coli* replicative vector which contains the mycophage L5 integrase gene required for attP/attB integration.	Arnold et al. 2018	Addgene #110096
pINIT-Cat *Mtb MmpS5L5*	FX cloning entry vector containing SapI-domesticated *Mtb* MmpS5L5 operon	This study	
pINIT-Cat *Mtb MmpS4L4*	FX cloning entry vector containing SapI-domesticated *Mtb* MmpS4L4 operon	This study	
pFLAG-attP-Kan	Single copy integrative vector, with L5 attP, but lacking L5 int. FX cloning compatible, with ccdB. Constitutive pmyctetO promoter and C-terminal 3x FLAG tag.	This study	
pFLAG-attP-Kan *Mtb-MmpS5L5*	pFLAG-attP-Kan containing a SapI-domesticated *Mtb* MmpS5L5 operon.	This study	
pFLAG-attP-Kan *Mtb MmpS5L5* N444K		This study	
pMINTF3	Single copy integrative vector, with mycobacteriophage L5 int and attP. FX cloning compatible, with ccdB. Constitutive pmyctetO promoter and C-terminal 3x FLAG tag.	This study	
pMINTF3 *Mtb MmpS5L5*	pMINTF3 containing a SapI-domesticated *Mtb* MmpS5L5 operon.	This study	
pMINTF3 *Mtb MmpL5*	pMINTF3 containing a SapI-domesticated *Mtb* MmpL5	This study	

Reagent or resource	Description	Source	Identifier
DNA constructs			
pMINTF3 *Mtb MmpS1-MmpL5*		This study	
pMINTF3 *Mtb MmpS2-MmpL5*		This study	
pMINTF3 *Mtb MmpS4-MmpL5*		This study	
pMINTF3 *Mtb MmpS5L5* Y277F		This study	
pMINTF3 *Mtb MmpS5L5* D849N		This study	
pMINTF3 *Mtb MmpS5L5* Y850F		This study	
pMINTF3 *Mtb MmpS5L5 ∆CC* (494–687)		This study	
pMINTF3 *Mtb MmpS5* K54A *MmpL5*		This study	
pMINTF3 *Mtb MmpS5* K140A *MmpL5*		This study	
pMINTF3 *Mtb MmpS5* K54A, K140A MmpL5		This study	
pMINTF3 *Mtb MmpS5* C137S *MmpL5*		This study	
pMINTF3 *Mtb MmpS5L5* M72F		This study	
pMINTF3 *Mtb MmpS5L5* K76G		This study	
pMINTF3 *Mtb MmpS5L5* A90F		This study	
pMINTF3 *Mtb MmpS5L5* F129L		This study	
pMINTF3 *Mtb MmpS5L5* Q150V		This study	
pMINTF3 *Mtb MmpS5L5* S189P		This study	
pMINTF3 *Mtb MmpS5L5* Q196M		This study	
pMINTF3 *Mtb MmpS5L5* Q196R		This study	
pMINTF3 *Mtb MmpS5L5* D417L		This study	
pMINTF3 *Mtb MmpS5L5* Y431D		This study	
pMINTF3 *Mtb MmpS5L5* N444K		This study	
pMINTF3 *Mtb MmpS5L5* N444E		This study	
pMINTF3 *Mtb MmpS5L5* F462M		This study	
pMINTF3 *Mtb MmpS5L5* Q707S		This study	
pMINTF3 *Mtb MmpS5L5* Q707L		This study	
pMINTF3 *Mtb MmpS5L5* I719V		This study	
pMINTF3 *Mtb MmpS5L5* M727Q		This study	
pMINTF3 *Mtb MmpS5L5* M764T		This study	
pMINTF3 *Mtb MmpS5L5* H832Y		This study	
pMINTF3 *Mtb MmpS5L5* N444K, Y331D		This study	
pMINTF3 *Mtb MmpS5L5* Y331D		This study	
pMINTF3 *Mtb MmpS5L5* Y331N		This study	
pMINTF3 *Mtb MmpS5L5* V193D		This study	
pMINTF3 *Mtb MmpS5L5* V902A		This study	
pMINTF3 *Mtb MmpS5L5* K598R		This study	
pMINTF3 *Mtb* MmpS4L4	Construct expressing MmpS4L4	This study	

Table S2. Oligonucleotide sequences used in this study

Oligo	Sequence	Description
P104	ATATATGCTCTTCTAGTCTAATGCGGACTTGGATTCCACTGGTCATCCTGG	Amplification of MmpS4L4 for insertion into FX-compatible vectors
P105	TATATAGCTCTTCATGCGCCGAGGCGGTCGCTGCTCATC	
P106	ATATATGCTCTTCTAGTATTGGAACTCTCAAGCGTGCCTG	Amplification of MmpS5L5 for insertion into FX-compatible vectors
P107	TATATAGCTCTTCATGCGACCAAGGCGAAGGTCCGTG	
P298	CGGACCTCTATTCACAGGGTACG	For constructing pFLAG-attP-Kan
P299	GCTGGAAGCACTCAACCTCG	
P300	GTACCCTGTGAATAGAGGTCCGGTGTCTCAAAATCTCTGATGTTAC	
P301	CGAGGTTGAGTGCTTCCAGCTCCTTCAACTCAGCAAAAG	
P352	TCCATGAAGGCGCAGATGCTG	For SapI domestication of the MmpS5L5 sequence
P353	CTTCATGGTCTGGATCATCTCGG	
P354	GCCTGCTGTTCGACACCCTGATC	
P355	CCATACCGATGGTGGTGCCAAC	
P366	ATCGTGCAAAGGACAGCTGC	For deletion of MmpS5 to create MmpL5 only construct
P367	ACTAGAAGAGCGGCCAACCAG	
P368	CTCGATCGGTTTGCCGTCCG	Use to remove MmpL5' coiled coil domain
P369	TCGTTCTATCTGCCTCCCGAGGTTTTC	
P465	TTACTTGTCATCGTCGTCCTTGTAATCACTAGTTTTGTAGAGCTCATCCATGCC	For constructing pMEXC3GF vector
P466	CATCATTGAGTTTAAACGGTCTCCAGC	
P577	CGGCCTGGTTGGCCGCTCTTCTAGTTTCGGCGTTGCCAAACGCTTC	PCR of MmpS1 with overhangs for replacing MmpS5
P578	TCGGCGCAGCTGTCCTTTGCACGATCATGCGGATTTCACCAGGCAACTG	
P579	CGGCCTGGTTGGCCGCTCTTCTAGTAGGATGATTTCGGTTAGCGGC	PCR of MmpS2 overhangs for replacing MmpS5
P580	CGTCGGCGCAGCTGTCCTTTGCACGATCATCCGGATGACACCTGGC	
P581	CGGCCTGGTTGGCCGCTCTTCTAGTCTAATGCGGACTTGGATTCCACTG	PCR of MmpS4 with overhangs for replacing MmpS5
P582	TCGGCGCAGCTGTCCTTTGCACGATCATGCGGACTTCACCAAGC	
P583	ATGATCGTGCAAAGGACAGCTG	For replacing MmpS5 with paralog. Used with P367
P623	GCGTTTTACGACCAGATGATCGG	Amplify MmpS5L5 without residues 2–106 for assembly with eblock
P624	TGCGGATTTCACAAAGCAGTAGGTC	
P625	GATCGAGGCGGTCACGTTCAC	Amplify MmpS5L5 without residues 115–205 for assembly with eblock
P626	CGATCATCTGGTCGTAAAACGCATGG	
P627	GTACTACACCATGTTCGGCGG	Amplify MmpS5L5 without residues 207–297 for assembly with eblock
P628	CACCTGCAGACTACGGTCG	
P629	CTACCGGACCAACTACAACGACCG	Amplify MmpS5L5 without residues 298–409 for assembly with eblock
P630	CGACTCCCGGTCCTGGCC	
P631	GAAACTGACCGAGAAATACAACCAGGAC	Amplify MmpS5L5 without residues 410–507 for assembly with eblock
P632	GGGTCAGCAGACCGACGAG	
P633	GTCATGACCGAAGACATCAACAACCTG	Amplify MmpS5L5 without residues 508–611 for assembly with eblock
P634	CTGGTGCCCTGCATGCTGATC	
P635	GGCCTGGAACAGTTCCTCTCG	Amplify MmpS5L5 without residues 612–703 for assembly with eblock
P636	GTCGATTCCGTCGAGGGTGTC	
P637	TTCATCATCATGCTGATCACCACCC	Amplify MmpS5L5 without residues 704–787 for assembly with eblock
P638	GCGTTGGAAGTCGGGATTGTC	
P639	GGCATCATCCGTGCGATGG	Amplify MmpS5L5 without residues 788–868 for assembly with eblock
P640	GATCAGGCAGAGTGCGGAGATTC	
P641	GCATGAAGAGCGGCCACC	Amplify MmpS5L5 without residues 869–964 for assembly with eblock
P642	GGTGTTGATTCCGGCGTGGATC	
P677	GTTCTCGGCTCGATGATCCACC	For sequencing MmpS5L5 from integrated constructs
P678	GCGAGTCAGTGAGCGAGGAAG	
P721	GCAACTCTTGTGCGACTCTTCTGAC	For construction of pMINTF3
P722	CGGTTCCTGGCCTTTTGCTG	
P723	ACATGTGAGCAAAAGGCCAGC	
P724	CGTATGCCCAGGTCAGAAGAGTC	
P725	GCCTGGTTGGCCGCTCTTCTAGTATTGG	For mutagenic PCR of MmpS5L5 with Mutazyme II
P726	CCTCGGTGGCCGCTCTTCATGC	
P812	ATTGTAAAACGACGGCCAGTAAAGAAAAACGTCCCGTTGGGAAC	PCR of *Mm atpE* for sequencing with M13F
P813	TTAAAATCCCGACCCCATACCAG	

Table S3. Cryo-EM data collection, refinement, and validation statistics

**Data collection and processing**	**MmpS5L5-BDQ** [EMD-53947, PDB 9RGB]	**MmpS5L5 (apo)** [EMD- 53941, PDB 9RFU]	**Monomeric MmpL5-acpM (+BDQ)** EMD-54511, PDB 9S2U]
Microscope	Krios Titan G4	Krios Titan G4	Krios Titan G4
Magnification	130,000	130,000	130,000
Voltage (kV)	300	300	300
Electron exposure ($e^{-}/Å^2$)	80	80	80
Defocus range (μm)	-0.6 to -2.2	-0.6 to -2.2	-0.6 to -2.2
Pixel size (Å)	0.955	0.955	0.955
Symmetry imposed	C1	C1	C1
Initial particle images (no.)	3,240,490	4,810,392	4,185,525
Final particle images (no.)	33,185	27,426	78,900
Map resolution (Å)	3.2	3.3	3.2
FSC (half-map) threshold	0.143	0.143	0.143
Map resolution range (Å)	2.7–3.7	2.9–3.8	2.7–3.7
**Model Refinement**			
Initial model used	AlphaFold2	AlphaFold2	AlphaFold2
Map sharpening *B* factor ($Å^2$)	-54.7	-65.2	-76.6
Model composition			
Non-hydrogen atoms	19,410	19,410	6,157
Protein residues	2,543	2,543	816
Ligands	6	6	0
Model-map scores			
CC (mask)	0.80	0.84	0.76
CC (box)	0.75	0.77	0.69
CC (peaks)	0.68	0.70	0.58
CC (volume)	0.79	0.83	0.75
Mean CC for ligands	0.66	0.70	–
R.m.s. deviations			
Bond lengths (Å)	0.005	0.005	0.007
Bond angles (°)	0.908	1.067	1.007
**Validation**			
MolProbity score	1.15	1.62	1.66
CaBLAM outliers (%)	1.00	1.16	1.24
Clashscore	3.64	7.85	7.04
Poor rotamers (%)	0.2	0	0
C-beta deviations (%)	0	0	0
EMringer score	1.47	1.78	1.20
Mean Q-score	0.63	0.64	0.51
Ramachandran plot			
Favored (%)	98.10	96.83	96.06
Allowed (%)	1.86	3.17	3.82
Disallowed (%)	0.04	0	0.12

SI References

1. J. García-Nafría, J. F. Watson, I. H. Greger, IVA cloning: A single-tube universal cloning system exploiting bacterial In Vivo Assembly. *Sci. Rep.* **6**, 27459 (2016).

2. E. R. Geertsma, “FX Cloning: A Simple and Robust High-Throughput Cloning Method for Protein Expression” in *DNA Cloning and Assembly Methods*, Methods in Molecular Biology., S. Valla, R. Lale, Eds. (Humana Press, 2014), pp. 153–164.

3. F. M. Arnold, *et al.*, A uniform cloning platform for mycobacterial genetics and protein production. *Sci. Rep.* **8**, 9539 (2018).

4. D. N. Woolfson, G. J. Bartlett, M. Bruning, A. R. Thomson, New currency for old rope: from coiled-coil assemblies to α-helical barrels. *Curr. Opin. Struct. Biol.* **22**, 432–441 (2012).

5. J. Kyte, R. F. Doolittle, A simple method for displaying the hydropathic character of a protein. *J. Mol. Biol.* **157**, 105–132 (1982).
